# Temperature controls organic carbon sequestration in a subarctic lake

**DOI:** 10.1038/srep34780

**Published:** 2016-10-06

**Authors:** Marttiina V. Rantala, Tomi P. Luoto, Liisa Nevalainen

**Affiliations:** 1 University of Helsinki, Department of Geosciences and Geography, P.O. Box 64, 00014 Helsinki, Finland; 2 University of Jyvaskyla, Department of Biological and Environmental Science, P.O. Box 35, 40014 Jyvaskyla, Finland

## Abstract

Widespread ecological reorganizations and increases in organic carbon (OC) in lakes across the Northern Hemisphere have raised concerns about the impact of the ongoing climate warming on aquatic ecosystems and carbon cycling. We employed diverse biogeochemical techniques on a high-resolution sediment record from a subarctic lake in northern Finland (70°N) to examine the direction, magnitude and mechanism of change in aquatic carbon pools prior to and under the anthropogenic warming. Coupled variation in the elemental and isotopic composition of the sediment and a proxy-based summer air temperature reconstruction tracked changes in aquatic production, depicting a decline during a cool climate interval between ~1700–1900 C.E. and a subsequent increase over the 20^th^ century. OC accumulation rates displayed similar coeval variation with temperature, mirroring both changes in aquatic production and terrestrial carbon export. Increase in sediment organic content over the 20^th^ century together with high inferred aquatic UV exposure imply that the 20^th^ century increase in OC accumulation is primarily connected to elevated lake production rather than terrestrial inputs. The changes in the supply of autochthonous energy sources were further reflected higher up the benthic food web, as evidenced by biotic stable isotopic fingerprints.

The Arctic is warming at an unprecedented rate^1^ with a myriad of influences on the ecological functioning and carbon balance of sensitive northern ecosystems^2^,^3^,^4^. Temperature has a fundamental control over northern landscapes through constraints on biological production and on material fluxes restrained by the freezing of terrestrial soils and prolonged snow and ice cover period. Even small shifts in temperature patterns thus have the potential to alter ecosystem productivity, community structure, and terrestrial and aquatic elemental cycles^3^,^5^. Recent research has demonstrated widespread increases in the concentrations of dissolved organic carbon (DOC) in lake waters across the Northern Hemisphere^6^,^7^, as well as increases in sediment organic content over the 20^th^ century^8^,^9^,^10^. These changes in aquatic carbon dynamics have profound effects on lake ecosystem functioning and metabolism^11^,^7^, yet no scientific consensus exists on the mediating role of the anthropogenic warming. Previous studies have evidenced a number of direct and indirect temperature controls over aquatic carbon pools, affecting aquatic production^12^, the generation and transport of organic matter from the terrestrial environment^4^, and abiotic and biotic mineralization in lake water^13^ and sediments^14^. Ecosystem responses are, however, highly heterogeneous and the climate influence may be superimposed on other human-induced environmental changes. Aside from climatic factors, changes in land-use^15^ and atmospheric deposition chemistry, including declines in sulfate deposition^6^ and increased nitrate deposition^10^, have been proposed as key drivers of recent change in aquatic OC dynamics. Deeper understanding of the underlying mechanisms, their complex interactions, and variability through time is wanting, hampering our ability to predict ecosystem responses to global change.

With this study we attempt to build towards a better understanding of the link between carbon dynamics and temperature in northern lakes by examining high resolution temporal variations in sediment OC pools prior to and under the recent warming in subarctic Lake Námmájávri at the Fennoscandian treeline (Fig. 1). Elemental (C, N) and stable isotopic (δ^13^C_OM_, δ^15^N_OM_) composition of the sediment were examined in connection with a proxy-based (fossil Chironomidae) summer air temperature reconstruction to track climate-driven changes in OC origin, concentrations and accumulation. Concomitant effects on carbon utilization in the benthic food web were investigated from the stable isotopic composition of fossil invertebrate (Cladocera) chitin. To estimate changes in solar radiation attenuation and lake water UV transparency, controlled by terrestrial OC input, the degree of UV absorbance of benthic Cladocera was assessed. We expected to find temperature-driven increase in the export of terrestrial OC in association with the 20^th^ century warming, contributing to an increase in the sediment carbon pools and affecting carbon utilization patterns and UV transparency in the lake. The results build towards an improved understanding of the influence of global change on aquatic carbon dynamics in northern lakes that constitute an integral element in the global carbon cycle.

## Results and Discussion

Our record depicts synchronous variations in temperature (Fig. 2) and the biogeochemical composition of sediment (Fig. 3) over the past 600 years, implying a tight connection between temperature and aquatic carbon pools. Comparable records of temperature variability over decadal time scales in northern Finland are sparse, yet the inferred temperature pattern from Lake Námmájávri is consistent with previous high-resolution proxy records from northwestern Finland^16^,^17^ and across the circumpolar arctic^1^. Previous studies have discerned warm temperature anomalies during the Middle Ages followed by a cool climate interval referred to as the ‘Little Ice Age’ (LIA) that marked the culmination of the Neoglacial cooling^1^,^18^,^19^. There is broad geographical variation in the timing of the LIA, ranging from the 13^th^ until the early 20^th^ century^19^, yet the distinct decline in temperatures at ~1700–1900 C.E. in the present record (Fig. 2) is consistent with earlier paleoenvironmental studies from Finland. Temperature minima were reached at around 1700 C.E. in southern Finland (60°N) and around 1900 C.E. in the eastern parts of the country (65°N)^18^. In agreement with our record (Fig. 2), Weckström *et al*.^17^ showed that low temperatures prevailed until the early 20^th^ century in northern Finland, followed by a distinct increase particularly over the latter half of the 20^th^ century. In concert, contemporary monitoring data from northern Finland (available 1960s onwards) depicts increasing mean annual temperature over recent decades^20^. The close correspondence of our record with earlier climate reconstructions and monitoring data together with good modern analogues (Fig. 2) suggest reliability of the inferred temperature pattern.

Bivariate correlations and multivariate analyses indicated a temperature coupling both with the sediment OC content and biogeochemical composition (Fig. 4, Table 1). We propose that the synchronous variation of temperature with the stable isotopic composition and C/N ratio of the sediment elucidates primarily changes in the relative contribution of autochthonous (i.e., produced within the lake) and allochthonous (i.e., derived from outside the lake) organic matter. Overall, the *δ*^13^C_OM_ values in Lake Námmájávri (mean −23.3‰) resemble those commonly associated with diffusion-limited benthic autochthonous production (typically > −25‰)^21^,^22^. Terrestrial plant matter is generally more ^13^C-depleted (~−27‰), but may yield values that overlap with the benthic signal^21^. In effect, the C/N ratio (mean 15) suggests a mixture of carbon from autotrophic production (~5–8) and from terrestrial sources (from 15 to >70)^23^. The low *δ*^15^N values (mean −0.4‰) in the sediment likely reflect the presence of N-fixing cyanobacteria^23^ that often dominate the benthic communities in shallow northern lakes^24^. Based on present limnological characteristics (Table 2), Lake Námmájávri represents a typical nutrient poor lake ecosystem dominated by allochthonous OC inputs^25^,^26^. The concentrations of DOC and colored dissolved organic matter (CDOM) in the lake are comparatively low (Table 2) but within the range observed from tundra lakes in the region^27^ and across the circumpolar arctic^28^,^24^. The optical characteristics of the DOM (Table 2), with high specific UV absorbance (SUVA) and relatively low fluorescence index (FI), imply terrestrial origins for the lake water carbon. Regardless of the low pelagic primary production, the stable isotope composition of the sediments suggests abundant benthic production, also common to clear and shallow arctic lakes where high light availability may support thriving periphytic growth^24^.The balance between allochthonous and autochthonous OC has implications for aquatic carbon balance, and unproductive northern lakes are commonly perceived as net heterotrophic ecosystems where community respiration exceeds primary production. While heterotrophy generally increases along the DOC gradient^25^,^29^, even lakes with very low DOC concentrations, such as the studied lake, may be net sources of carbon to the atmosphere^30^.

A positive correlation between the inferred temperature and the *δ*^13^C_OM_ (Fig. 4, Table 1) imply increased autochthonous contribution to the sediment OM with increasing temperatures. The *δ*^15^N_OM_ further showed a pattern very similar to that of *δ*^13^C_OM_, aside from few samples at the bottom of the core (Fig. 3). Similarly, the C/N ratio varied in parallel with the temperature (Figs 2, 3, 4), though no statistically significant correlation was found (*r *= −0.41, *p *= 0.05). The synchronous variations likely mirror increased aquatic primary production under warming temperatures, resulting in progressive ^13^C and ^15^N enrichment of the sediment OM as the lake water C and N pools become increasingly enriched in the heavier isotopes^8^, and in declining C/N values with increasing algal contribution^23^. The elevated *δ*^13^C_OM_ and *δ*^15^N_OM_ values may additionally be related to weakened physiological fractionation against the heavier isotopes as the lake water C and N pools become depleted upon biological uptake^21^. Conversely, the negative excursion in both *δ*^13^C_OM_ and *δ*^15^N_OM_ and elevated C/N values during the coolest LIA phase between ~1700–1900 C.E. imply lower autochthonous contribution to the sediment OM.

**Figure 1:**
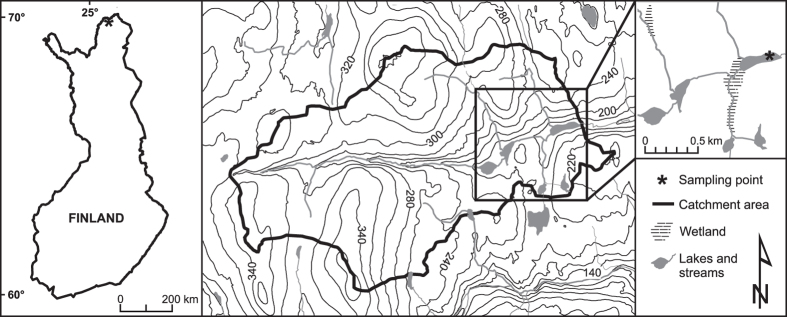
Location of Lake Námmájávri (69°49′N, 26°56′E) in northern Finland, catchment area, topography, and surface-water features. The map is modified from National Land Survey of Finland open data (Basic Map Series, General Map 05/2016) that are licensed under a Creative Commons Attribution 4.0 International License. A copy of the license is available at http://creativecommons.org/licenses/by/4.0/. The map was compiled using ArcGIS 10.3.1 (http://desktop.arcgis.com/en/arcmap/) and CorelDRAW Graphics Suite X7 (http://www.coreldraw.com/rw/product/graphic-design-software/).

**Figure 2:**
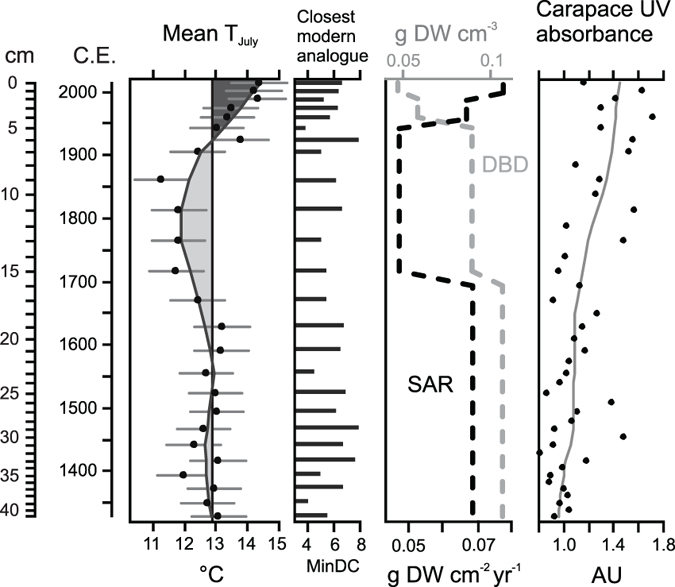
Inferred temperature, sediment physical properties, and UV absorbance in benthic Cladocera in Lake Námmájávri. Mean July air temperature is reconstructed from fossil Chironomidae assemblages, displayed with sample-specific errors (eSEP) and results from modern analogue technique (MAT) identifying samples with ‘no close’ analogues in the modern calibration data (cut-off value 8.2). Additionally, sediment accumulation rate (SAR), dry bulk density (DBD), and *Alona affinis* carapace UV absorbance are presented.

**Figure 3:**
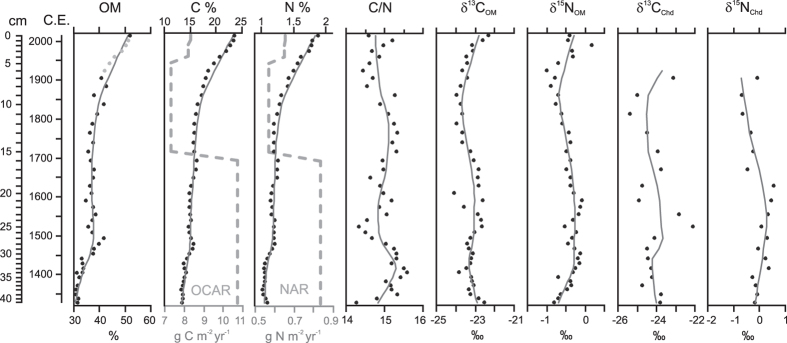
Sediment biogeochemical properties in Lake Námmájávri. Sediment organic matter (OM) is based on loss-on-ignition. Values at 1–6 cm (grey) were calculated from sediment carbon (C) based on linear regression between OM and C (*r*^2^ = 0.97) in the surface sediments of a regional lake set^27^ due to low sample residue (<20 mg) after ignition. For sediment C and nitrogen (N), both elemental contents and annual accumulation rates (OCAR, NAR) are presented. Stable carbon and nitrogen isotope values (*δ*^13^C_OM/Chd_, *δ*^15^N_OM/Chd_) derive from sediment bulk OM and fossil cladoceran (Chydoridae) remains. Grey lines indicate locally weighted scatter plot smooth (lowess, span 0.4).

Changes in the length of the ice-free period seem a plausible explanation for the observed patterns as aquatic primary production is strongly controlled by light availability^31^ that in northern lakes is restricted by the extensive ice-cover period. In concert, paleolimnological and instrumental records have evidenced lengthening of the ice-free period in arctic lakes over the 20^th^ century^32^, resulting in marked shifts in aquatic community structure and production stimulated by changes in habitat and resource availability^5^,^33^. A few studies have elucidated concurrent sedimentary responses, showing parallel increases in sediment OC content^8^,^9^ or OC accumulation rates^10^ with increasing lake productivity. While endorsing the mediating role of climate warming^8^,^10^, these studies have underlined the importance of a mechanism other than temperature, related primarily to increased nutrient loading from the terrestrial environment or via atmospheric deposition. The biogeochemical composition of the sediments in Lake Námmájávri yielded little evidence of increased terrestrial contribution (Fig. 3). Elevated UV absorbance values of benthic Cladocera throughout the 19^th^ and 20^th^ centuries (Fig. 2) further imply high lake water UV transparency, giving no support to increased concentrations of terrestrial colored DOC that acts as a key UV attenuator in northern lakes^34^. The increasing *δ*^15^N_OM_ in Lake Námmájávri over the 20^th^ century neither gives support to fertilization by atmospheric reactive N that generally yields an opposite trend^8^,^9^,^35^. Terrestrial retention of N deposition may explain the absence of such an influence as the studied lake has a large catchment to lake area ratio (Fig. 1).

**Figure 4:**
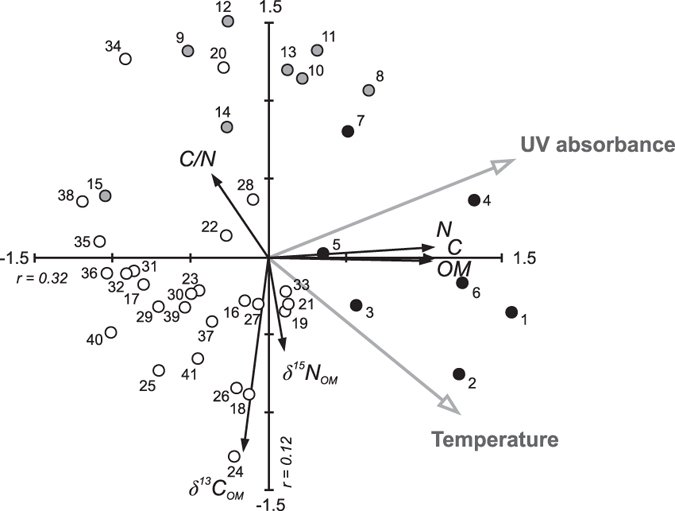
Redundancy analysis (RDA) triplot summarizing variation in the sediment geochemistry in Lake Námmájávri across environmental gradients. Inferred mean July air temperature and UV transparency (carapace UV absorbance) were included as explanatory variables that together account for 39.9% of the total variation in the sediment geochemistry. Circles represent samples at different sediment depths, falling between ~1300–1700 C.E. (white), ~1700–1900 C.E. (grey), and ~1900 C.E.–present (black). Eigenvalues for the first two environmental gradients are presented.

**Figure 5:**
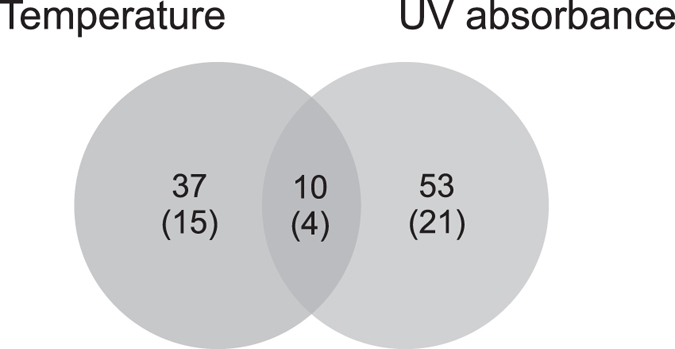
Venn diagram showing the fraction (%) of variance in the sediment geochemistry explained by mean July air temperature and carapace UV absorbance. The values in brackets indicate explained fraction of total variance in the response data.

Elemental OC content (as weight percent) is a relative measure that does not take into account compositional changes nor accommodate for changes in sediment delivery, as also evidenced by the disparity between OC content and OCAR in Lake Námmájávri (Fig. 3). This divergence, however, allows us to further explore the apparent link between temperature and sediment OC pools. OCAR is derivative of sediment accumulation rate and dry bulk density, both of which have varied in the studied sediment profile related to post-burial processes, compositional changes, and external forcing. The declining dry density throughout the sediment profile (Fig. 2) is likely partially attributable to sediment compaction in the deeper layers. Moreover, dry density often decreases along with increasing OM content^36^, and thus the increase in sediment OM towards the surface may in part diminish the apparent annual OCAR. While the high OC, N and OM in the topmost sediments are likely overestimated owing to incomplete mineralization, degradation of sediment OM generally occurs during the first few years after deposition^37^, and thus cannot account for the multidecadal increase in all three proxies. Gälman *et al*.^37^ suggested that most of the decline in sediment C and N content will take place within the first five years following deposition after which only minor changes are likely to occur. Differential degradation of C and N may also distort the C/N values in surface sediments^37^, and at least the topmost C/N value is likely underestimated (relative to older sediments with higher N loss). Aside from the above factors that reduce bulk dry density (and consequently annual elemental accumulation rates) towards the surface, the OCAR pattern is largely controlled by changes in sediment accumulation rate (Figs 2 and 3). Periods of elevated accumulation rates were connected with the warmer temperatures of the pre-LIA and the 20^th^ century, suggesting direct climate influence on aquatic production or indirect catchment-mediated influence. Furthermore, the latter increase is associated with significantly lower minerogenic component as suggested by the coincident increase in the sediment OC content (Fig. 3). In line with the high UV transparency (Figs 2 and 4), this likely implies that the 20^th^ century increase in OCAR is primarily driven by enhanced aquatic production. The highest OC accumulation rates over the medieval warm period and early LIA, coupled with high minerogenic component in the sediment and low UV absorbance values, suggest combined climate influence mediated both via elevated aquatic production and increased terrestrial carbon export. The coolest LIA interval was characterized by elevated UV absorbance values that likely reflect lower terrestrial DOC export. The strong connection between UV transparency and sediment organic content suggested by the data analyses (Figs 4 and 5, Table 1) thus seems to derive from both climate-driven changes in terrestrial carbon export and in aquatic production.

Changes in terrestrial vegetation may lag behind temperature fluctuations by several decades^38^ and we suggest that the divergence in sediment OC content and accumulation rates between the two warm periods (Fig. 3) may similarly reflect a delayed catchment (soil, vegetation) response to the warming climate. Accordingly, the changes in sediment biogeochemistry over the 20^th^ century likely reflect initial temperature stimulus on aquatic production, driven primarily by improved light availability under lengthened ice-free period. The following catchment response is likely to not only increase the export of OC but also of allochthonous nutrients that may further promote lake production^12^,^39^. It may be argued that the benthic-dominated community in Lake Námmájávri is more responsive to the initial temperature influence on the length of the growing season (rather than the subsequent allochthonous nutrient influence) as benthic growth is primarily controlled by light availability^31^, although recent studies have evidenced a strong nutrient control also on periphyton in oligotrophic arctic lakes^40^. It seems likely that the distinctly elevated OC accumulation rates during the termination of the medieval warm period and the early LIA reflect both catchment and lake responses to prolonged warmth. A minor increase in the C/N ratio over the latter half of the 20^th^ century (Fig. 3) could indicate a gradual increase in terrestrial OC export, although no parallel changes were observed in the absorbance values nor in the minerogenic content of the sediment. Cory *et al*.^13^ proposed that the fate of DOC in shallow arctic freshwaters is largely controlled by solar influence on photochemical and biological mineralization which could also partly explain the inferred low levels of allochthonous OC over the 20^th^ century. Lengthened ice-free period subjects DOC to increased photochemical degradation, potentially supplemented by increasing UV intensities over recent decades^41^. Overall, the cumulative impact of the rapid 20^th^ century warming could be manifested for years to come, albeit a variety of physical and chemical processes may obscure the intrinsic responses of terrestrial carbon stocks to temperature increase^4^. We also cannot exclude synchronous or synergistic variations in hydrological controls that determine whether enhanced accretion of terrestrial OM is reflected in aquatic OC pools^42^, although previous studies from Finland have not evidenced consistent changes in humidity over the 20^th^ century^18^,^43^. Changes in catchment hydrology and flow pathways may also interact here, affecting the aromaticity and recalcitrance of the transported carbon as well as concomitant nutrient export. For instance, an increase in deep flow paths relative to shallow may result in higher flux of low aromaticity carbon^27^,^44^ that will have a less pronounced influence on solar radiation attenuation in the water column.

The temperature fluctuations and consequent changes in the relative supply of autochthonous OC were also mirrored at the consumer level, as evidenced by the stable isotopic composition (*δ*^13^C_Chd_, *δ*^15^N_Chd_) of benthic Cladocera (family Chydoridae) that constitute a central link in aquatic carbon transfer from primary producers to higher trophic levels. The *δ*^13^C_Chd_ and *δ*^15^N_Chd_ followed a pattern very similar to the isotopic composition of the bulk sediment (Fig. 3), suggesting that the Chydoridae primarily feed unselectively on periphyton and detritus in the lake. The chydorids were slightly ^15^N-enriched compared to the sediment (mean difference 0.4 ± 0.3‰), which could be attributable to the more efficient excretion of ^14^N relative to ^15^N during metabolization^45^. Considering an offset of around −7 to −9‰ between the chitinous exoskeletons and whole bodies and a taphonomic effect of up to +5‰ 46,47, the N isotopic composition of the Chydoridae is likely to be even more enriched in ^15^N (estimated at around +4‰ relative to measured *δ*^15^N_Chd_ values). Typical trophic enrichment for N between the consumer and food source is +2–4‰^48^, and thus the *δ*^15^N values of the cladoceran diet are relatively close to those of the bulk sediment. For *δ*^13^C, the offset between exoskeletons and whole individuals, taphonomic processes, and trophic fractionation generally have a minimal effect^46^,^49^, although acid treatment has been shown to increase the values by approximately +2‰ relative to untreated remains^46^. The isotopic composition of the Chydoridae was generally slightly^13^C-depleted relative to the bulk sediment (mean difference −0.9 ± 0.7‰) and considering the effect of the acid treatment the negative offset is likely to be slightly larger. ^13^C-depletion in consumers relative to their diet has been observed in earlier studies^50^ and may be related to the accumulation of more ^13^C depleted organic compounds (such as lipids), or to preferential utilization of terrestrial detritus.

**Figure 6:**
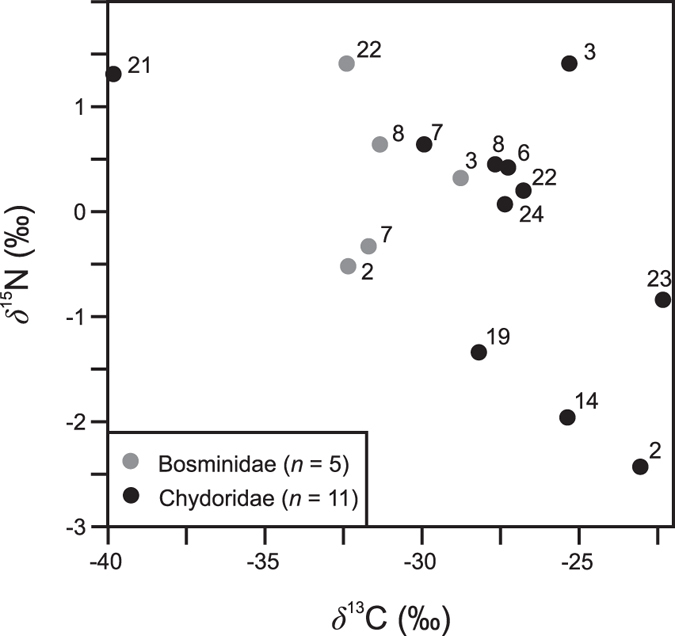
Stable isotopic composition (*δ*^13^C, *δ*^15^N) in benthic and planktonic Cladocera (Chydoridae, Bosminidae) in the surface sediments of lakes in northern Finland.

The stable isotopic composition of benthic Cladocera was further assessed from the surface sediments of subarctic lakes across a treeline transect in northern Finland (information on site characteristics provided in Rantala *et al*.^27^). The *δ*^13^C_Chd_ and *δ*^15^N_Chd_ in the regional lake set were strongly correlated with the stable isotopic composition of the sediment OM (*r *= 0.92, *p *= 0.001 and *r* = 0.87, *p *= 0.001, respectively). The surface sediment *δ*^13^C_OM_ and *δ*^15^N_OM_ in the lakes mirror primarily variation in the contribution of carbon from terrestrial sources and aquatic benthic production to the sediment carbon pools^27^. Thus, the data suggest that spatial variation in *δ*^13^C and *δ*^15^N in the sediment, and in the benthic fauna, across diverse catchment types is strongly connected to terrestrial influence. In Lake Námmájávri, DOC and CDOM concentrations were among the lowest in the regional lakes set, which likely explains why the stable isotopic fingerprints in the sediment profile seem to track primarily in-lake processes (i.e., aquatic productivity). To allow comparison with the pelagic zone, the stable isotopic composition (*δ*^13^C_Bos_, *δ*^15^N_Bos_) of planktonic Cladocera (family Bosminidae) was analyzed from the surface sediments, showing marked stability in contrast with the benthic community (Fig. 6), which likely indicates selective feeding on phytoplankton. A previous Holocene record^51^ as well as a contemporary study on zooplankton feeding patterns^22^ from northern Finland similarly suggested reliance on phytoplankton by Bosminidae. In accordance, the *δ*^13^C_Bos_ values in the surface sediments were generally lower when compared with the *δ*^13^C_Chd_ and correspond with those associated with phytoplankton^21^. The *δ*^15^N_Bos_ values were slightly elevated relative to the *δ*^15^N_Chd_ values, which may be attributed to the utilization of benthic cyanobacteria as a food source by the benthic taxa^24^.

Warming temperatures have been shown to result in wide-spread regime shifts in aquatic communities^33^, often reflected most readily at the base of the food web, but with potential cascading effects across all trophic levels^12^,^31^. Our results indicate little changes in the feeding patterns and trophic position of the benthic Cladocera over the study period, yet suggest that changes in the source of carbon in the sediment organic matter pool will be reflected in the cladoceran diet. Under extreme environmental conditions, changes in the supply of food items with differing nutritional quality may have an influence on the growth and reproduction of the benthic fauna and, consequently, on reliant components of the food web. The changes in lake productivity suggested by our record may have also implication for aquatic carbon balance, and the observed increase in autochthonous production rather than allochthonous OC export in the lake under the anthropogenic warming may imply an improved carbon sink over the 20^th^ century. This does not, however, exclude contemporaneous increase in the release of methanogenic carbon which may be promoted by the lengthening of the growing season, warmer water temperatures, altered mixing regimes, and increase in the availability of organic substrate for methanogenesis^52^.

## Conclusions

Our record depicts elevated OC accumulation rates in connection with the medieval warm period and the anthropogenic warming. The biogeochemical carbon indices suggest that the former increase in sediment OC sequestration reflects both direct and indirect climate effect on aquatic production and on the export of terrestrial OC into the lake. The increasing OC accumulation over the 20^th^ century seems to derive primarily from elevated autochthonous production, as suggested by the benthic stable isotopic signatures, high organic content of the sediment, and high UV transparency in the water column. While recent studies have evidenced similar patterns in lake production attributed primarily to atmospheric N inputs, our results suggest that temperature influence on ice phenology and light availability for aquatic primary production is a key driving mechanism in the studied lake. We propose that the high drainage ratio of the lake may dampen the influence of altered atmospheric deposition chemistry. Our data provide little evidence of recent increases in the terrestrial export of OC observed widely in lakes across the Northern Hemisphere over the past decades. This may, however, reflect delayed catchment response to the warming temperatures, possibly supported by increased OC degradation in the lake water under lengthened ice-free period and increasing solar irradiance. The present record contributes to our understanding of temperature influence on shallow oligotrophic northern lake ecosystems, and suggest that past temperature fluctuations have altered the ecological structure and functioning of the studied lake.

## Materials and Methods

### Study area

Lake Námmájávri (69°49′N, 26°56′E) is a small and shallow oligotrophic lake situated in the treeline ecotone in northern Finnish Lapland (Fig. 1) some 60 km poleward of the northernmost limit of continuous pine (*Pinus sylvestris*) treeline. Mountain birch (*Betula pubescens* ssp. *czerepanovii*) woodland dominates at lower elevations in the topographically diverse fell terrain to an elevation of around 250 m. The lake lies in a depression surrounded by steeply sloping fells to the north and south, draining water from a large catchment area (900 ha) with an overall altitudinal gradient of around 200 m. The basin is hydrologically open, with a narrow inlet and an outlet stream, and is connected to two smaller lakes upstream (Fig. 1). The surrounding soils at the valley floor are partly waterlogged. Mountain birch woodland covers the sheltered lower fell slopes being replaced by dwarf shrub heats, shrubs and lichens higher in the fells. The lake is situated within a granite gneiss complex east of the Lapland granulite belt, comprising mostly acidic gneissic granites and hornblende gneisses. The catchment is characterized by a mosaic of glacial tills of differing thickness dotted by bedrock outcrops at higher elevations. Subarctic climate prevails and the region is affected by the fluctuating influence of temperate continental and arctic/temperate maritime air masses. Mean annual temperature, mean July temperature, and mean annual precipitation in the area are −1.3 °C, 13.1 °C, and 430 mm, respectively, based on observations between years 1981–2010 at the nearby Kevo meteorological station (N 69°45′, E 27°00′)^53^. The study region is situated in the zone of discontinuous permafrost. Aside from reindeer grazing, the lake is not subjected to direct anthropogenic influence. Limnological and biogeochemical data from a regional lake set across the treeline in northern Finland (including Lake Námmájávri) were additionally used in this study as modern reference data. Details on catchment characteristics, limnology and sediment geochemistry are provided in Rantala *et al*.^27^.

### Sampling and biogeochemical analyses

A sediment core was retrieved in July 2014 at the center of Lake Námmájávri (Fig. 1) at a water depth of 1.7 m using a Limnos gravity corer. The 42-cm sediment profile consisting of homogenous fine-detritus gyttja was subsampled at intervals of 1 cm (resulting in 42 samples) in the field and stored in Minigrip^®^ plastic bags at 4 °C prior to the analyses. The sediments were analyzed for loss-on-ignition following standard methods^54^, with wet sediment samples dried at 105 °C for 15 hours and subsequently ignited at 550 °C for 4 hours to determine organic matter (OM) content. Where the sample residue fell below 20 mg (n = 5), OM was estimated based on C content using linear regression between sediment C and OM (*r*^2^ = 0.97) in the surface sediments of lakes in the region^27^. To confirm the absence of carbonates, the samples were further ignited at 950 °C for 2 hours^54^. The elemental content (C, N) and stable isotopic composition (*δ*^13^C_OM_, *δ*^15^N_OM_) of sediment OM were analyzed from freeze-dried and homogenized sediments. The analyses were run in duplicate and mean values were used in the data analyses. On average, duplicate measurements of δ^13^C_OM_ differed by 0.4‰ (three samples exceeding 1.0‰), δ^15^N_OM_ by 0.1‰ (one sample exceeding 0.5‰), C by 0.1‰, and N by 0.01‰. Determination of the stable isotopic composition of carbon and nitrogen in chitinous cladoceran (Chydoridae and Bosminidae) exoskeletons (*δ*^13^C_Chd/Bos_, *δ*^15^N_Chd/Bos_) was done following Perga^47^. The sediments were heated with 10% potassium hydroxide (KOH) for 30 minutes to remove organic matter coating from the remains, followed by treatment with 1 M HCl to remove any carbonate coating. The samples were rinsed thoroughly on a 100 μm filter cup with Milli-Q^®^ water, and cladoceran remains (carapaces and headshields) were picked with fine forceps under stereomicroscope at 40× magnification. The analysis was performed at intervals of 2 cm (every other sample), hand picking approximately 500‒1000 Chydoridae remains to yield a sample mass of ≥0.2 mg. The samples consisted mostly of *Alona affinis* remains, supplemented with low amount of remains of other benthic taxa to ensure adequate sample mass. The topmost 7 cm were excluded due to scarcity of remains. Stable isotopic analysis on Chydoridae remains was additionally performed on the surface sediments of 11 lakes in the regional lake set^27^ where remains were found in sufficient numbers. From five lakes, also pelagic Bosminidae were found in high numbers in the surface sediments and approximately 1000 remains (mostly *Eubosmina longispina*) were picked and analyzed separately. The stable isotope ratios are expressed relative to standard as the delta notation *δ* = R_sample_/R_standard_ − 1 × 1000, where R equals ^13^C/^12^C for carbon and ^15^N/^14^N for nitrogen. The respective standards used were Vienna Pee Dee Belemnite (VPDB) and atmospheric nitrogen (AIR). Analytical precision (expressed as standard deviation) was determined based on internal laboratory working standards for bulk sediment samples (birch leaves, n = 19) and cladoceran remains (fish muscle tissue, n = 10). For the bulk sediment samples, standard deviations between the replicate reference standards were 0.4‰ (*δ*^13^C_OM_), 0.3‰ (*δ*^15^N_OM_), 0.2% (C) and 0.03% (N). For the chitinous remains, the corrensponding values were 0.4‰, 0.05‰, 0.2% and 0.1%. The C content of the chydorid exoskeletons was also assessed to ensure that the changes in *δ*^13^C_Chd_ are not related to C degradation^46^ (no correlation between the two).

### Chironomidae-based temperature reconstruction

Subsamples for fossil chironomid analysis were prepared applying standard methods^55^. The wet sediment was gently sieved through a 100-μm mesh and the residue was examined using a Bogorov counting chamber under a stereomicroscope at 32–40× magnification. Larval head capsules were extracted with fine forceps and mounted permanently with Euparal on microscope slides. Faunal identification was performed under a light microscope at 400× magnification based on Brooks *et al*.^55^. The minimum chironomid head capsule number per sample was set to 50, and the analysis was performed at 2-cm intervals apart from the topmost 7 cm that were analyzed at 1-cm intervals (resulting in 25 samples). The chironomid-based mean July air temperature reconstruction used the regional expanded Fennoscandian calibration model (weighted-averaging partial least squares, WA-PLS) combining several datasets^56^,^57^,^58^,^59^. The temperature gradient in the training set varies from 7.9 to 17.6 °C. The 2-component model currently includes 180 lakes and 129 taxa having a bootstrapping cross-validated coefficient of determination (r^2^_boot_) of 0.86, a root mean squared error of prediction (RMSEP) of 0.86 °C, and a maximum bias of 0.77 °C. Lake Námmájávri was removed from the calibration set to avoid a bias in the reconstruction. A direct gradient analysis, canonical correspondence analysis (CCA), was used to assess the strength of temperature in determining chironomid community composition in the training set. The ratio of the first constrained eigenvalue (λ_1_) to the second unconstrained eigenvalue (λ_2_) indicates the relative significance of a specific variable in explaining the cumulative variance in the species data. In the present training set, λ_1_/ λ_2_ was >1 (1.178), suggesting that temperature is an important ecological determinant for the chironomid distribution and may be used in quantitative inference models^60^. Sample-specific errors (eSEP) were estimated using bootstrapping cross-validation (999 iterations). Using the modern analogue technique (MAT), the cut-level of the 5^th^ percentile of all squared chord-distances in the modern calibration data were determined. These distances were then compared to the distance between each fossil assemblage and its most similar assemblage in the modern dataset and used to define ‘no close’ analogues.

### Spectral absorbance measurements

UV absorbance was measured from fossil cladoceran (Chydoridae, *Alona affinis*) carapaces following the protocol established by Nevalainen & Rautio^61^. This included sieving of the sediment through a 100-μm mesh, extracting the carapaces under a binocular microscope, and measuring UV absorbance (305 and 340 nm) of the remains using a specifically designed adapter in a UV-VIS spectrophotometer. Seven carapaces were measured from each down-core subsample, and the average of absorbance values was used after highest and lowest absorbance values were omitted. Carapace UV absorbance is previously deemed to be dependent on UV transparency of the habitat giving indications on past changes in underwater UV exposure^34^.

### Chronology

**Figure 7:**
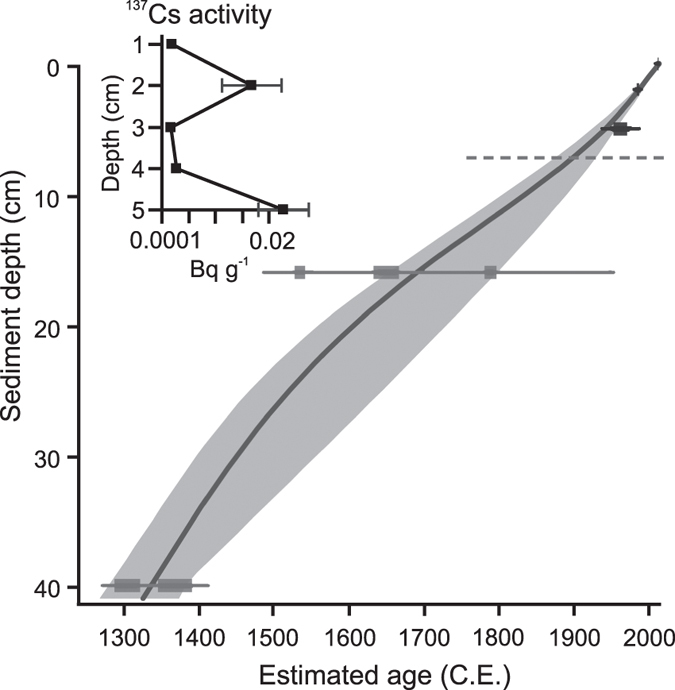
Age-depth model for Lake Námmájávri. Smooth spline (smooth level 0.4) was employed including age estimates from AMS ^14^C (grey) and ^137^Cs (black) analyses. Grey envelopes represent 95% confidence intervals. ^14^C age at 7 cm (shaded line) represents “modern” (<200 B.P.). Additionally, ^137^Cs activities with sample-specific error estimates are presented.

Core chronology was established based on accelerator mass spectrometry (AMS) radiocarbon (^14^C) dating of four terrestrial macrofossils (Table 3) and radiocesium (^137^Cs) dating of bulk sediment (Fig. 7). ^14^C ages at 16 and 24 cm were not in chronological order and only the former was included owing to higher sample mass. The sample at 7 cm was modern, i.e., <200 B.P, and was added as a reference point in the final chronology. Peaks in ^137^Cs activity at 2 cm and 5 cm were associated with the Chernobyl nuclear accident in 1986 and nuclear weapons testing in 1940–60s. Lead (^210^Pb) dating was additionally employed, but no reliable age estimates were obtained owing to low activities (≤0.2 Bq g^−1^) typical for northern lakes. The radiocarbon dates were calibrated using Clam 2.2^62^ that was also used to build an age-depth model. The calibration was based on the northern hemisphere terrestrial curve IntCal13^63^ and the age-model was constructed using smooth spline (smoothing level 0.4), 2 σ confidence intervals, and 10 000 iterations. Point age estimates for each depth were based on the weighted mean of the entire date distribution of the models^62^. To obtain crude estimates of OC accumulation rates (OCAR) and nitrogen accumulation, mean sedimentation rates between the dated intervals were multiplied by dry bulk density and fractional C and N contents that were similarly averaged over the age intervals. The resulting four time intervals correspond roughly with 1300–1700, 1700–1950, 1950–1990 C.E., and 1990 C.E.–present.

### Numerical analyses

Constrained direct gradient analysis, redundancy analysis (RDA), was employed to summarize variation in the geochemical composition of the sediment (OM, OC, N, *δ*^13^C_OM_ *δ*^15^N_OM_) and to detect relationships to the chironomid-inferred mean July air temperature and lake water UV transparency (UV absorbance of benthic Cladocera). Variance partitioning analysis (VPA) with Monte Carlo permutation tests (permutations set at 999) were further used to assess which fraction of the variance in the geochemical data is explained by the two predictor variables. Additionally, pairwise correlations between the variables were assessed with parametric correlation tests. False discovery rate (FDR) using the Benjamini-Hochberg procedure was employed to correct for multiple comparisons, setting the q-value at 0.01. Prior to the analyses, Shapiro-Wilk and skewness were assessed together with visual inspection of the data to check for data normality. The predictor variables and response variables that departed from normality were square root transformed. Response variables were centered and standardized for the ordination. The topmost sample (0–1 cm), representing the past ~10 years, was excluded from the data analyses to account for the effects of incomplete mineralization.

## Additional Information

**How to cite this article**: Rantala, M. V. *et al*. Temperature controls organic carbon sequestration in a subarctic lake. *Sci. Rep.* **6**, 34780; doi: 10.1038/srep34780 (2016).

## Figures and Tables

**Table 1:** Statistically significant (FDR adjusted significance level = 0.04, two-tailed test) pairwise correlations between sediment biogeochemical indices and chironomid-inferred mean July air temperature and carapace UV absorbance, with correlation coefficients (black) and *p*-values (grey).

	C	N	OM	δ^13^C
Mean T_July_	0.52 **0.010**	0.55 **0.006**	0.55 **0.006**	0.44 **0.032**
UV absorbance	0.68 **0.000**	0.68 **0.000**	0.65 **0.000**	—

**Table 2:** Catchment characteristics and limnological properties of Lake Námmájávri.

Catchment	*Unit*		Limnology	*Unit*	
Latitude	°N	69°49.860	DOC	mg L^−1^	1.8
Longitude	°E	26°56.883	a_320_	m^−1^	4
Altitude	m	169	SUVA	mg C L^−1^ m^−1^	2.4
Catchment slope	m	211	FI		1.2
Lake area	ha	3	Color	Pt mg^−1^	5
Catchment area	ha	901	Total phosphorus	μg L^−1^	5.9
Drainage ratio		283	Total nitrogen	μg L^−1^	138
Mean air T_annual_	°C	−1.3	Chlorophyll-a	μg L^−1^	0.56
Mean air T_July_	°C	13.1	pH		7.8

DOC = dissolved organic carbon, a_320_ = absorption coefficient at 320 nm, SUVA = specific UV absorbance, FI = fluorescence index. Details on analytical techniques are provided in Rantala *et al*.^27^.

**Table 3:** AMS radiocarbon dating results.

Depth (cm)	Material	^14^C age (BP)	Cal. age (BP)	Cal. age 2σ interval (BP)
7	twig	<200	—	—
16	leaf fragment	255	258	151‒295
24	twig	235	411	286‒463
41	leaf fragment	635	623	575‒681

Samples at 7 cm (“modern”) and 24 cm (intercrossing with the age at 16 cm, latter included due to higher sample mass) were omitted from the final age-depth model.
